# UDP-4-Keto-6-Deoxyglucose, a Transient Antifungal Metabolite, Weakens the Fungal Cell Wall Partly by Inhibition of UDP-Galactopyranose Mutase

**DOI:** 10.1128/mBio.01559-17

**Published:** 2017-11-21

**Authors:** Liang Ma, Omar Salas, Kyle Bowler, Maor Bar-Peled, Amir Sharon

**Affiliations:** a Department of Molecular Biology and Ecology of Plants, Tel Aviv University, Tel Aviv, Israel; b Complex Carbohydrate Research Center, University of Georgia, Athens, Georgia, USA; c Department of Plant Biology, University of Georgia, Athens, Georgia, USA; Duke University

**Keywords:** *Botrytis cinerea*, UDP-4-keto-6-deoxyglucose, UDP-galactopyranose mutase, fungal cell wall, fungal nucleotide sugar metabolism, galactofuranose, metabolic intermediate

## Abstract

Can accumulation of a normally transient metabolite affect fungal biology? UDP-4-keto-6-deoxyglucose (UDP-KDG) represents an intermediate stage in conversion of UDP-glucose to UDP-rhamnose. Normally, UDP-KDG is not detected in living cells, because it is quickly converted to UDP-rhamnose by the enzyme UDP-4-keto-6-deoxyglucose-3,5-epimerase/-4-reductase (ER). We previously found that deletion of the *er* gene in *Botrytis cinerea* resulted in accumulation of UDP-KDG to levels that were toxic to the fungus due to destabilization of the cell wall. Here we show that these negative effects are at least partly due to inhibition by UDP-KDG of the enzyme UDP-galactopyranose mutase (UGM), which reversibly converts UDP-galactopyranose (UDP-Gal*p*) to UDP-galactofuranose (UDP-Gal*f*). An enzymatic activity assay showed that UDP-KDG inhibits the *B. cinerea* UGM enzyme with a *K*_*i*_ of 221.9 µM. Deletion of the *ugm* gene resulted in strains with weakened cell walls and phenotypes that were similar to those of the *er* deletion strain, which accumulates UDP-KDG. Gal*f* residue levels were completely abolished in the Δ*ugm* strain and reduced in the Δ*er* strain, while overexpression of the *ugm* gene in the background of a Δ*er* strain restored Gal*f* levels and alleviated the phenotypes. Collectively, our results show that the antifungal activity of UDP-KDG is due to inhibition of UGM and possibly other nucleotide sugar-modifying enzymes and that the rhamnose metabolic pathway serves as a shunt that prevents accumulation of UDP-KDG to toxic levels. These findings, together with the fact that there is no Gal*f* in mammals, support the possibility of developing UDP-KDG or its derivatives as antifungal drugs.

## IMPORTANCE

Nucleotide sugars are donors for the sugars in fungal wall polymers. We showed that production of the minor sugar rhamnose is used primarily to neutralize the toxic intermediate compound UDP-KDG. This surprising finding highlights a completely new role for minor sugars and other secondary metabolites with undetermined function. Furthermore, the toxic potential of predicted transition metabolites that never accumulate in cells under natural conditions are highlighted. We demonstrate that UDP-KDG inhibits the UDP-galactopyranose mutase enzyme, thereby affecting production of Gal*f*, which is one of the components of cell wall glycans. Given the structural similarity, UDP-KDG likely inhibits additional nucleotide sugar-utilizing enzymes, a hypothesis that is also supported by our findings. Our results suggest that UDP-KDG could serve as a template to develop antifungal drugs.

## INTRODUCTION

The fungal cell wall is a robust yet flexible structure composed of different polysaccharide structures, including chitin, glucans, mannans, and a range of different proteins that are often glycosylated (1, 2). The cell wall is vital for fungal survival and proper development because it provides the fungal cell with mechanical strength, which contributes to the ability of the fungus to withstand osmotic changes and other stresses (3, 4). The vital role of the fungal wall and the absence of primary wall elements in mammals make the fungal wall a prime target for antifungal drugs. Indeed, several antifungal drugs target the wall, including polyoxins and echinocandins that target synthesis of chitin and beta-1,3-glucan, respectively (5).

The biosynthesis and remodeling of fungal cell walls involve concerted actions of enzymes that use a range of activated nucleotide sugars as donors to synthesize specific glycan components that are necessary for cell wall formation. For example, the synthesis of chitin is mediated by chitin synthases that utilize UDP-*N*-acetylglucosamine as a substrate to synthesize linear chitin (2), UDP-glucose is utilized by glucan synthases to produce various glucans (2), and GDP-mannose is used by mannosyltransferases to produce a range of proteins that are heavily decorated with mannose residues (manno-proteins) (6, 7). Other nucleotide sugars common in most fungi are UDP-galactopyranose (UDP-Gal*p*) and UDP-galactofuranose (UDP-Gal*f*), which are used for the synthesis of diverse types of polysaccharides (8) and glycoconjugates (9). In addition to the abundant sugars that are found in the cell walls of all fungal species, several types of minor UDP-sugars are found only in certain species, for example UDP-rhamnose (10, 11), UDP-xylose (12), and UDP-glucuronic acid (13).

Recently, two genes involved in the synthesis of UDP-4-keto-6-deoxyglucose (UDP-KDG) and UDP-rhamnose were identified (14) and analyzed in two plant-pathogenic fungi, *Botrytis cinerea* and *Verticillium dahliae* (10, 11). Deletion of the second gene (*er*) that encodes the enzyme UDP-4-keto-6-deoxyglucose-3,5-epimerase/-4-reductase (ER) resulted in accumulation of UDP-KDG and reduced the ability of both fungi to infect plants (10, 11). Interestingly, deletion of the *B. cinerea er* gene (*bcer*) also caused developmental defects that were associated with weakening of the cell wall; however, the mechanism leading to wall defects and reduced virulence remained unclear.

Many transporters and glycosyltransferases can accommodate multiple substrates and could be potentially inhibited by analogues of their nucleotide sugars (15–20). Given the structural similarity of UDP-KDG with many nucleotide sugars, we postulated that the toxicity of UDP-KDG might be due to inhibition of glycosyltransferases that utilize nucleotide sugars to assemble glycans and glycoconjugates. It is also possible that UDP-KDG affects enzymes that are involved in conversion of one nucleotide sugar to another. Accordingly, we searched for UDP-sugar-utilizing enzymes that could be putative targets of UDP-KDG. Here we report on the identification of the UDP-galactopyranose mutase (UGM) enzyme, which reversibly converts UDP-galactopyranose to UDP-galactofuranose, as a target of UDP-KDG. We show that UDP-KDG inhibits *B. cinerea* UGM, which results in reduced amounts of cellular and wall Gal*f*, and weakening of the fungal cell wall.

## RESULTS

### Inhibition of UDP-galactopyranose mutase by UDP-KDG.

**FIG 1:**
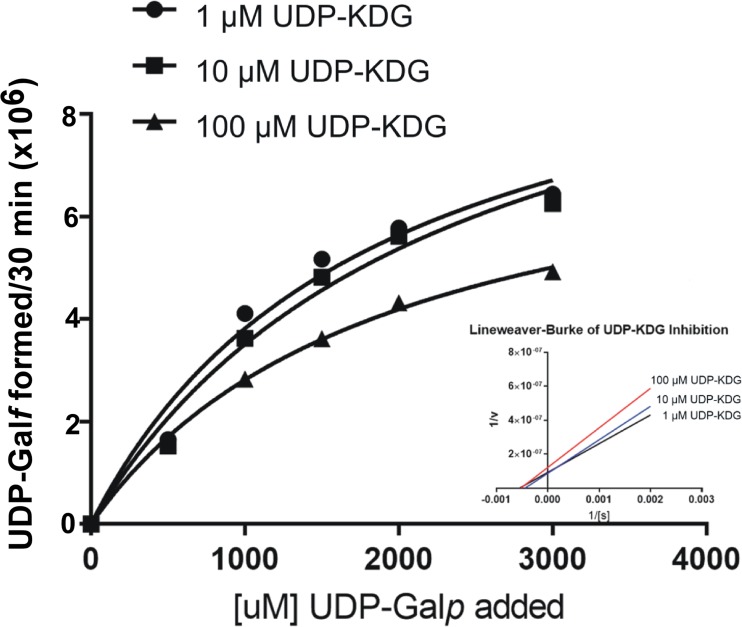
UDP-KDG inhibits *B. cinerea* UDP-galactopyranose mutase. Kinetic analyses were performed with increasing UDP-Gal*p* concentrations (0.1, 0.5, 1.0, 1.5, 2.0, and 3.0 mM). For the inhibition assays, the reactions remained the same as for the kinetic analyses with the addition of various concentrations of UDP-KDG (1, 10, and 100 μM).

A possible mechanism by which UDP-KDG affects cell wall integrity is inhibition of UDP-sugar-utilizing enzymes that are involved in the synthesis of other UDP-sugars like UDP-glucose, UDP-galactose (UDP-Gal), and UDP-*N*-acetylglucosamine. To test this possibility, we subjected the *Botrytis cinerea* genome to a BLAST search for homologues of proteins involved in nucleotide sugar metabolism. Among several candidate genes that were found, we selected BC1G_09363 (NCBI), which potentially encodes UDP-galactopyranose mutase (UGM), for further analysis. This gene was expressed in *Escherichia coli*, and enzymatic activity of the recombinant protein was determined. Figure S1 in the supplemental material shows that the BC1G_09363 protein is active as a UDP-galactopyranose mutase, transforming UDP-galactopyranose to UDP-galactofuranose in the presence of NADPH and flavin adenine dinucleotide (FAD) (Fig. S1C). This interconversion catalysis was further determined by tandem mass spectrometry (MS/MS) analyses (Fig. S1G to I); hence, this gene product was named UDP-galactopyranose mutase. Kinetic analyses of enzyme activity showed that the *K*_*m*_ of the enzyme to UDP-Gal*p* is 395 µM and that it was strongly inhibited by UDP-KDG with a *K*_*i*_ value of 221.9 µM (Fig. 1).

10.1128/mBio.01559-17.1 FIG S1  Identification of UDP-galactopyranose mutase (UGM) from *Botrytis*. LC-MS/MS analyses of enzyme activity of recombinant *Botrytis* proteins, expressed and purified from *E. coli*. After enzyme assays, reactions were separated by HILIC column and detected by mass spectrometry (MS) and tandem mass spectrometry (ms/ms). The UGM enzyme is active when reacted with UDP-galactopyranose (UDP-Gal*p*), FAD, and NADPH (C), forming a product eluting at ~11.5 min with *m/z* of 565.02, while no activity was observed without the cofactors (B) or with negative-control protein (D), *E. coli* expressed and purified recombinant UDP-Glc 4,6-dehydratase. (E) Similar UGM reaction condition as in panel C with elution of the product at ~11.5 min with *m/z* of 565.0 [M-H]^−^, and also showing that UDP-Glc is not interfering with UGM activity (F). Further ms/ms analyses of the 565 peak eluting at 11.5 min had a characteristic mass fragment (I) 322.9 of UDP-hex. Download FIG S1, TIF file, 2.7 MB. Copyright © 2017 Ma et al. 2017 Ma et al. This content is distributed under the terms of the Creative Commons Attribution 4.0 International license.

### *bcugm* and *bcer* deletion strains share similar phenotypes.

To investigate whether the effects of UDP-KDG are mediated by inhibition of *B. cinerea* UGM (BcUGM), we generated *bcugm* deletion mutants and compared their phenotypes to those of a previously reported Δ*bcer* strain, which does not produce UDP-rhamnose but accumulates UDP-KDG (10). The following strains were used in the analyses: *bcugm* deletion strain Δ*bcugm2b1* (abbreviated Δ*ugm*) strain, *bcer* deletion strain Δ*bcer9a2* (abbreviated Δ*er*) strain, *bcer bcugm* double deletion strain Δ*bcer* Δ*bcugm1c* (abbreviated Δ*er* Δ*ugm*) strain, *bcugm* overexpression in the background of Δ*er* strain Δ*bcer*::OE*bcugm5a* (abbreviated Δ*er*::OE*ugm*) strain, and *bcugm* complementation strain Δ*bcugm*+*bcugm6a* (abbreviated Δ*ugm*+*ugm*) strain.

### (i) Mycelial growth.

**FIG 2:**
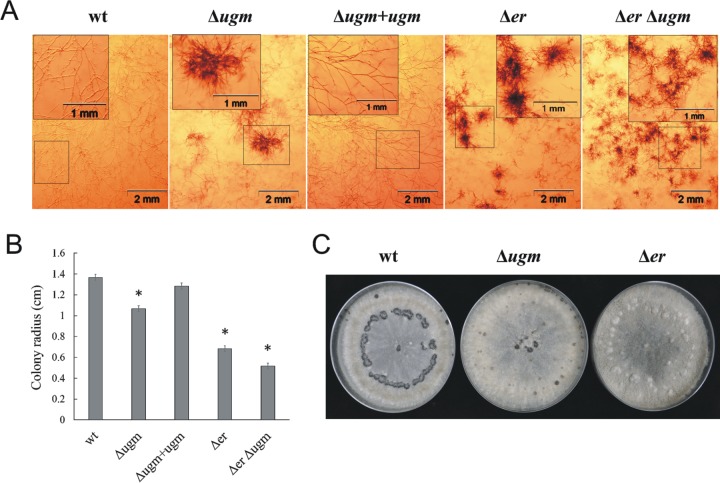
The Δ*ugm* strain and the Δ*er* strain share similar developmental and growth abnormalities. Cultures were initiated by placing a 5-μl droplet containing 500 spores in the center of each plate. (A) Fungi were cultured in CD medium under continuous light for 3 days. Cultures were inspected using a stereomicroscope, and images were captured. Photographs show hyphal organization of the wild-type (wt) strain, Δ*ugm* strain, Δ*ugm*+*ugm* complementation strain, Δ*er* strain, and Δ*er* Δ*ugm* double deletion strain. (B) Fungal growth in CD medium. Data are the mean radii plus standard deviations (SD) (error bars) for three 3-day-old colonies. Values that are statistically significantly different (*P* < 0.01 by two-tailed Student’s *t* test) from the wild-type values are indicated by an asterisk. (C) Fungi were cultured on PDA medium in complete darkness for 11 days. Photographs show sclerotia formation of the wt, Δ*ugm*, and Δ*er* strains.

Colonies of the Δ*ugm* strain developed irregular mycelial clumps and had reduced radial growth rate compared with wild-type colonies (Fig. 2A and B). Both of these phenotypes were also observed in the Δ*er* strain; however, they were more severe than in the Δ*ugm* strain, as demonstrated by more-intense formation of mycelial clumps and more-pronounced growth retardation. Furthermore, these phenotypes were even more severe in the Δ*er* Δ*ugm* mutant strain, in which both the *bcer* and *bcugm* genes were deleted (Fig. 2A and B).

### (ii) Sclerotium formation.

After 11 days of incubation in dark, the Δ*ugm* strain produced aberrant sclerotia that were much smaller and less melanized than sclerotia produced by the wild-type strain (Fig. 2C). Similar to the growth-associated changes, the defects in sclerotium formation in the Δ*er* strain were more pronounced than in the Δ*ugm* strain.

### Pathogenicity.

**FIG 3:**
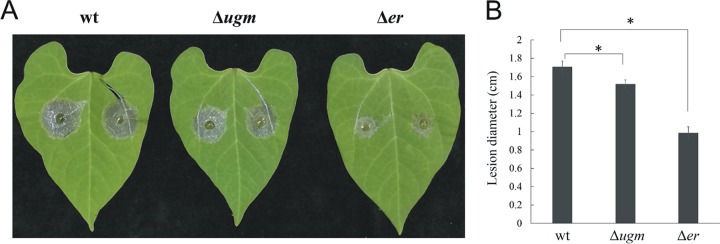
Mildly reduced virulence in the Δ*ugm* strain. Bean (*Phaseolus vulgaris*) leaves were inoculated with 7.5-μl droplets of a spore suspension containing 10^5^ spores/ml. Inoculated plants were incubated in a humid chamber at 22°C with light. (A) Typical lesions 3 dpi. (B) Average lesion size 3 dpi. Data are the means plus SD for eight lesions. Values that are statistically significantly different (*P* < 0.01) by two-tailed Student’s *t* test from the wild-type values are indicated by a bracket and asterisk.

Infection of bean leaves with spores of the Δ*ugm* strain resulted in well-developed lesions at 3 days postinfection. These lesions were intermediate in size compared to the larger lesions of the leaves inoculated with the wild-type strain and the smaller lesions caused by the Δ*er* strain (Fig. 3).

The similarity of the phenotypes for the Δ*ugm* and Δ*er* strains supports the possibility that UDP-KDG inhibits BcUGM *in vivo*. The more-severe appearance of all of the examined phenotypic characteristics in the Δ*er* strain indicates that UDP-KDG possibly has additional targets other than BcUGM, a hypothesis that is further supported by the even more severe phenotype of the Δ*er* Δ*ugm* double mutant.

### The Δ*ugm* strain has cell wall defects.

**FIG 4:**
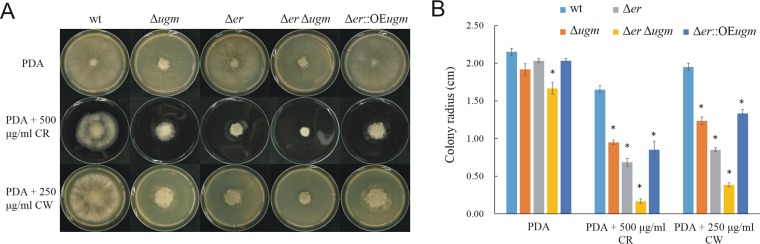
The Δ*ugm* strain displays cell wall defects. Colonies were initiated by placing a 5-μl droplet containing 500 spores in the center of each plate, and the plates were incubated at 22°C. PDA plates contain 500 μg/ml Congo red (CR) or 250 μg/ml calcofluor white (CW) as cell wall stressors. (A) Colony spreading at 3 dpi. (B) Average colony radii measured at 3 dpi. Data are the mean radii ± SD for three colonies. Values that are statistically significantly different (*P* < 0.01 by two-tailed Student’s *t* test) from the wild-type values are indicated by an asterisk.

To test for possible alterations of the cell wall in the Δ*ugm* strain, fungi were cultured on media containing the cell wall stressors calcofluor white (CW) and Congo red (CR) that perturb cell wall assembly by binding to chitin (21, 22). Growth of the Δ*ugm* strain was markedly reduced on media containing either 500 µg/ml CR or 250 µg/ml CW, concentrations that only slightly inhibit growth of the wild-type strain (Fig. 4). These results show that the Δ*ugm* strain has a weakened cell wall, thus highlighting the importance of UGM for cell wall integrity. The effects of the cell wall stressors on growth of the Δ*er* or Δ*er* Δ*ugm* strains were even stronger than on growth of the Δ*ugm* strain, which is in accordance with the more-severe developmental defects observed in these strains. Overexpression of the *bcugm* gene in the Δ*bcer* background (Δ*er*::OE*ugm* strain) partially compensated for the defects, further supporting the notion that UGM is inhibited by UDP-KDG in the Δ*er* strain (Fig. 4).

**FIG 5:**
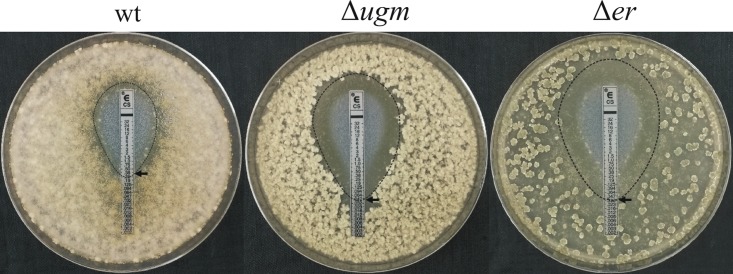
The Δ*ugm* and Δ*er* strains are more susceptible to caspofungin. Etest strips impregnated with a gradient of caspofungin were placed on Gamborg-glucose agar plates containing 10^4^ spores/ml of the different strains and cultured for 5 days at 22°C. The zone of inhibition is outlined by the black dashed line, and the MIC is read as the point where the ellipse of the zone of inhibition intercepts the test strip (black arrow).

Caspofungin is an antifungal drug (a semisynthetic echinocandin) that inhibits the activity of (1→3)-β-d-glucan synthase and thereby disturbs the integrity of the fungal cell wall (23). To test for possible synergism between inhibition of UGM and caspofungin, we cultured the strains on media and evaluated their sensitivity to caspofungin by using Etest strips (24). At 5 days postinoculation (dpi), the wild-type strain developed confluent growth in the presence of caspofungin and the MIC was 0.38 μg/ml. The MIC for the Δ*ugm* strain was significantly less (0.047 μg/ml), and a clear zone of inhibition was observed (Fig. 5). Similarly, the Δ*er* strain also showed a wider zone of inhibition compared to the wild type, with a caspofungin MIC (0.032 μg/ml) lower than that of the Δ*ugm* strain (Fig. 5).

Collectively, these results provide evidence that loss or inhibition of BcUGM affects sensitivity of the fungus to cell wall-targeting compounds, confirming that the cell wall integrity of both strains is compromised.

### Galactofuranose formation is inhibited in the Δ*er* strain.

**FIG 6:**
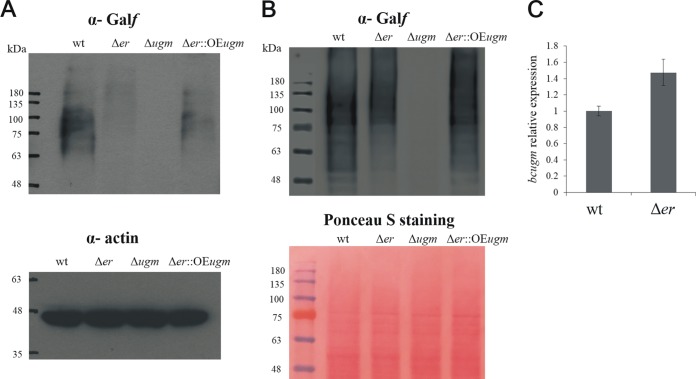
Galactofuranose (Gal*f*) formation is reduced in the Δ*er* strain. (A) Western blot to detect Gal*f* content in total cellular proteins. One hundred micrograms of isolated proteins was loaded for each strain. (Top) Anti-Gal*f* (α -Gal*f*) L10-1 monoclonal antibodies were applied to detect Gal*f* content. (Bottom) Antiactin monoclonal antibody was applied as a control for protein amount in total cellular protein samples. (B) Western blot to detect Gal*f* content in cell wall proteins. Fifty micrograms of isolated proteins was loaded for each strain. (Top) Anti-Gal*f* L10-1 monoclonal antibodies were applied to detect Gal*f* content. Signals of samples on a single blot membrane were detected using a MicroChemi imaging system (DNR; Bio-Imaging Systems, Israel). The positions of molecular mass markers (in kilodaltons) of a merged protein ladder in the unlabeled leftmost lane are indicated to the left of the gel. (Bottom) The Ponceau S protein staining was used for a loading control. (C) Comparison of *bcugm* expression compared to *bcgpdh* expression.

UGM enzymes catalyze the interconversion of UDP-Gal*p* and UDP-Gal*f*. Since UDP-Gal*f* is the donor for Gal*f* residues, comparison of Gal*f* content in glycoproteins can be used to estimate the level of UDP-Gal*f*. Consequently, if BcUGM is inhibited by UDP-KDG, the amount of the sugar residue Gal*f* that is incorporated into cell wall glycans should be reduced in the Δ*er* strain. Fungal cell wall glycoproteins usually contain Gal*f* in both N-linked glycans and O-linked glycans, which play important roles in protein stability, secretion, and localization (9). In order to determine the relative Gal*f* content, we extracted both cell wall proteins and total cellular proteins from different fungal strains and detected the Gal*f* epitopes by Western blotting with anti-Gal*f* monoclonal antibody L10-1 (25). Protein bands that mainly varied in size between 48 and 200 kDa were recognized in total cellular proteins and cell wall protein samples extracted from the wild-type strain (Fig. 6A and B). No signal was detected in samples of the Δ*ugm* strain, although equal amounts of proteins were loaded. The lack of antibody reaction with proteins extracted from the Δ*ugm* strain confirms the following. (i) The gene studied encodes a functional UGM. (ii) No alternative metabolic routes can form UDP-Gal*f*. (iii) UDP-Gal*f* is the sugar donor for the synthesis of Gal*f*-containing glycoproteins in *B. cinerea*. Our working model suggests that UDP-KDG accumulation in the Δ*er* strain has an inhibitory effect on overall formation of UDP-Gal*f*. Accordingly, the Δ*er* strain should contain reduced amounts of Gal*f* in the glycoproteins. In agreement with this scenario, the anti-Gal*f* signals in the Δ*er* strain were significantly reduced in total cellular proteins (Fig. 6A) as well as in cell wall proteins (Fig. 6B) compared with the Gal*f* amounts in protein extracts from the wild-type strain. Analysis of gene expression showed 1.5-fold-higher expression level of the *bcugm* gene in the Δ*er* strain (Fig. 6C), ruling out the possibility that reduction of Gal*f* level in glycoproteins is due to reduced levels of *bcugm* gene expression. Collectively, these results support our biochemical analyses that the reduced UGM enzymatic activity in the Δ*er* strain results from inhibition of BcUGM by UDP-KDG.

## DISCUSSION

Nucleotide sugars are precursors of primary metabolic pathways yielding diverse structures of glycoproteins, polysaccharides, and glycolipids (10). The fungal cell wall is composed primarily of glycans, including structural sugar polymers, such as chitin and glucans, and a range of glycoproteins that are decorated with various types of sugars. The roles of specific sugars within large polymers or glycoproteins are not easy to decipher, because the sugar may act as a structural element, it may act to stabilize an enzyme that is glycosylated, or it may have a role as a recognition epitope. In a previous study, we elucidated a two-step metabolic pathway of UDP-rhamnose production in *B. cinerea* (10). The first enzyme (UDP-glucose-4,6-dehydratase [DH]) converts UDP-glucose to produce the intermediate metabolite UDP-KDG, which is quickly converted to UDP-rhamnose by ER (see Fig. S2A in the supplemental material). A loss-of-function strain in which the entire UDP-rhamnose pathway was deleted was indistinguishable from the wild-type strain, and we could not infer any harmful consequence for the lack of rhamnose in the fungus. However, blocking of the second step of the pathway by deletion of the *bcer* gene led to multiple developmental defects, which were associated with weakening of the cell wall. We showed that the defects were due to accumulation of the intermediate metabolite UDP-KDG, which as far as we can tell is not used for metabolism other than UDP-rhamnose formation. Hence, the rhamnose metabolic pathway might serve as a shunt that prevents accumulation of UDP-KDG to toxic levels by converting it to UDP-rhamnose.

10.1128/mBio.01559-17.2 FIG S2  The proposed biosynthetic pathway for the formation of UDP-rhamnose in fungi. (A) In fungi, two separate enzymes involved in the enzymatic transformation of UDP-glucose to UDP-rhamnose. The enzyme DH (UDP-glucose-4,6-dehydratase) converts UDP-glucose to UDP-KDG (UDP-4-keto-6-deoxyglucose). Subsequently, the enzyme ER (UDP-4-keto-6-deoxyglucose-3,5-epimerase/-4-reductase) converts UDP-KDG to UDP-rhamnose. (B) UDP-KDG is nonenzymatically found in three different states (4-keto, hydrated, and keto-enol derivative), with approximately 9:1 in its preferred hydrated form. In fungi, UDP-glucose 4-epimerae (UGE) reversibly catalyzes UDP-glucose to UDP-Gal*p* (UDP-galactopyranose). UDP-galactopyranose mutase (UGM) reversibly catalyzes UDP-Gal*p* to UDP-Gal*f* (UDP-galactofuranose). We propose that in fungi lacking ER activity, the UDP-KDG accumulation leads to inhibition of UGM. Download FIG S2, TIF file, 2.5 MB. Copyright © 2017 Ma et al. 2017 Ma et al. This content is distributed under the terms of the Creative Commons Attribution 4.0 International license.

In humans, certain metabolic disorders are caused by the accumulation of toxic intermediate compounds when the product of an enzyme cannot be used by a downstream enzyme in the metabolic network (26). For example, classical galactosemia happens with infants who lack galactose-1-phosphate uridyl transferase activity (27), leading to the accumulation of galactose-1-phosphate, which functions as a potent competitive inhibitor of phosphoglucomutase to disrupt the glycolytic pathway (28). In microorganisms, accumulation of certain metabolic intermediates can also bring about toxic effects. For instance, accumulation of sugar-phosphates due to disruption of sugar metabolism have been shown to be highly toxic in *Mycobacterium tuberculosis* (29, 30). In the fungal system reported herein, deletion of *bcer* led to defects that result from accumulation of the toxic intermediate metabolite UDP-KDG, while lack of rhamnose had no effect on the fungus. Searching the JGI MycoCosm database (http://genome.jgi.doe.gov/programs/fungi/index.jsf) using *B. cinerea* DH (BcDH) as a query revealed that the 100 highest ranking fungi for blastp top hits also contained BcER homologues, further indicating that the *er* gene tends to coexist with the *dh* gene, probably as a safeguard to prevent accumulation of UDP-KDG.

Given the structural similarity of UDP-KDG with other nucleotide sugars, we reasoned that UDP-KDG might interfere with certain nucleotide sugar-utilizing enzymes. Among several potential targets, we found that UDP-KDG inhibits BcUGM. It is possible that the inhibition is due to the nature of UDP-KDG to exist in 4-hydrated form (Fig. S2B), but further studies, including cocrystallography of the BcUGM with UDP-KDG will be required to determine the nature of the interaction of BcUGM with UDP-KDG.

To evaluate whether inhibition of BcUGM by UDP-KDG is associated with the developmental defects of the Δ*er* strain, we produced *bcugm* deletion strains and compared their phenotypes with the phenotype of the Δ*er* strain. Overall, the phenotypic characteristics of the Δ*ugm* strain resembled those of the Δ*er* strain, including growth inhibition, developmental changes, impaired virulence, and weakening of the cell wall; however, the phenotypic changes of the Δ*ugm* strain were milder than those of the Δ*er* strain. Coupled with *in vitro* inhibition of BcUGM by UDP-KDG, these results suggested that *in vivo* inhibition of BcUGM by UDP-KDG accounts at least partly for the developmental defects of the Δ*er* strain. The consistently weaker effect in the Δ*ugm* strain suggests that UDP-KDG might have additional targets other than BcUGM. This possibility was further supported by the phenotypic characteristics of the Δ*er* Δ*ugm* double deletion strain that were more severe than the phenotypic characteristics of the Δ*er* strain and the milder phenotypic characteristics in the Δ*er*::OE*ugm* double deletion mutant.

Since UGM facilitates the conversion of UDP-Gal*p* to UDP-Gal*f*, which is the donor for the Gal*f* incorporated into glycans and glycoconjugates, the levels of Gal*f* are indicative of UGM activity in the cell. We estimated the relative levels of Gal*f* in the mutants by Western blot analysis using monoclonal antibodies that recognize Gal*f*-containing epitopes. In accordance with the expected inhibition of BcUGM, Gal*f* levels were markedly reduced (but not abolished) in the Δ*er* strain. Transcript levels of the *bcugm* gene in the Δ*er* strain were slightly higher than in the wild-type strain, further confirming that reduced Gal*f* levels are due to inhibition of the BcUGM enzyme. In addition, Gal*f* content in the Δ*er*::OE*ugm* strain was higher than that in the Δ*er* strain, indicating that the inhibition of Gal*f* production by UDP-KDG can be mitigated by overexpressing *bcugm*. Taken together, these results show that UDP-KDG inhibits the enzymatic activity of BcUGM not only *in vitro* but also *in vivo*, resulting in decreased Gal*f* levels and a weakening of the fungal cell wall.

Gal*f*-containing structures are important for survival and pathogenicity in many pathogenic microorganisms. These Gal*f*-containing structures include the lipopolysaccharides (LPS) O antigens of Gram-negative bacteria, extracellular or capsular polysaccharides of a variety of Gram-positive and Gram-negative bacteria, surface glycoconjugates of some protozoan parasites, as well as fungal cell wall and secreted molecules (8, 31). Because Gal*f* production depends on UGM, changes or defects in UGM activity lead to changes in Gal*f* that can affect fungal growth and virulence. For example, the *Aspergillus niger* Δ*ugm* mutant exhibits increased sensitivity to cell wall-perturbing agents (32), and in *Aspergillus nidulans*, the *ugm* gene is necessary for conidiation, hyphal growth, and morphogenesis, and it also affects antifungal drug sensitivity (33, 34). In addition, disruption of *ugm* in *Aspergillus fumigatus* results in weakened cell wall and likely attenuated virulence in mice (25, 35). Similarly, disruption of *bcugm* in *B. cinerea* resulted in weakened cell wall and other developmental defects. Since there is no Gal*f* in mammals and higher plants, Gal*f* biosynthetic pathways have been explored as attractive targets for the development of antibacterial drugs (36–41). The amino acid sequence conservation between prokaryotic and eukaryotic UGM is less than 15% (8), indicating that fungal and bacterial UGM enzymes are quite divergent. Because of these structural differences, it is expected that substrate binding mechanisms also differ in the fungal and bacterial UGM (42), and therefore, inhibitors of bacterial UGM are not expected to be efficient in fungi. Besides, all of these inhibitors were identified by *in vitro* enzymatic assays, which may not predict their entry into and true efficacy in living cells. Since UDP-KDG inhibits BcUGM both *in vitro* and *in vivo*, it might be possible to develop antimetabolites that mimic UDP-KDG as a UGM-targeting antifungal drug. Another way might be to identify compounds that inhibit nucleotide-rhamnose synthase (NRS)/ER enzyme as a way to force accumulation of toxic intermediates.

Although the Δ*ugm* strain phenocopied the Δ*er* strain in various aspects, the phenotype differed in some specific features such as reduced spore size and delayed spore germination that were not observed in the Δ*er* strain (Fig. S3). Considering the difference from the Δ*ugm* strain, the remaining low levels of Gal*f*, or the specific salvage pathway stimulated by severe cell wall defects in the Δ*er* strain, may underlie the unique characteristics of the Δ*ugm* strain. On the other hand, the Δ*er* strain displayed a more-debilitated phenotype than the Δ*ugm* strain did, meaning that BcUGM is not sufficient to account for all of the adverse effects brought about by UDP-KDG. In order to test whether UDP-KDG can inhibit other cell wall-synthesizing enzymes besides UGM, both *in vitro* and *in vivo* inhibition assays have yet to be performed.

10.1128/mBio.01559-17.3 FIG S3  Delayed spore germination and reduced spore sizes in the Δ*ugm* strain. (A) Spore germination in PDB medium. Spores were diluted to 5 × 10^4^/ml, 30 μl of spore suspension was incubated at 22°C, and the spore germination rate in each sample was determined by using an inverted microscope (Olympus). A total of 50 ± 10 spores were contained in each field of view to exclude the effects of different densities on germination. Four replicates were used for each sample. (B) Spore size measurement. Spore suspensions were examined using a microscope, and data are the mean major axis length ± SD for 40 spores. Values that are statistically significantly different (*P* < 0.01 by two-tailed Student’s *t* test) from the value for the wild type are indicated by an asterisk. Download FIG S3, TIF file, 2.7 MB. Copyright © 2017 Ma et al. 2017 Ma et al. This content is distributed under the terms of the Creative Commons Attribution 4.0 International license.

Drug synergy can result when drugs target the same or functionally related pathway but through different targets (43). The higher sensitivity of the Δ*ugm* and Δ*er* strains to cell wall inhibitors suggested that a UGM-targeting drug might be applied in combination with antifungal drugs that target other cell wall-synthesizing pathways, a notion that was supported by its potentiating the sensitivity of the mutants to caspofungin. Because caspofungin inhibits the enzyme 1,3-β-d-glucan synthase, the combination of deletion of *bcugm* with caspofungin resulted in a synergistic effect. Interestingly, despite partial inhibition of BcUGM in the Δ*er* strain, the sensitivity of the Δ*er* strain to caspofungin was slightly higher than that of the Δ*ugm* strain, possibly due to inhibition of multiple targets by UDP-KDG. These results demonstrate the antifungal activity of UDP-KDG, but more research will be necessary to determine the true potential of UDP-KDG as a lead molecule for antifungal therapies.

## MATERIALS AND METHODS

### Fungi and medium.

*Botryotinia fuckeliana* (de Bary) Whetzel (=*Botrytis cinerea*) strain B05.10 and derived transgenic strains were cultured on potato dextrose agar (PDA) (Acumedia, Lansing, MI) at 22°C with continuous fluorescent light supplemented with near-UV light. Routine culture or growth assays were carried out in potato dextrose broth (PDB) (Acumedia, Lansing, MI), Czapek Dox (CD) medium (0.3% NaNO_3_, 0.05% KCl, 0.05% MgSO_4 _⋅ 7H_2_O, 0.001% FeSO_4_ ⋅ 7H_2_O, 0.1% K_2_HPO_4_ ⋅ 3H_2_O, 2% sucrose [pH 7.3]), or GB medium (Gamborg B5 including vitamin mixture; Duchefa Biochemie, Haarlem, the Netherlands) supplemented with 2% glucose (GB5-Glc).

### Cloning and expression of *bcugm.*

Bacterial codon-optimized *B. cinerea* gene BC1G_09363 (potentially encodes UDP-galactopyranose mutase [BcUGM]) was synthesized (GenScript). For recombinant protein production, the gene was PCR amplified and cloned into pET28b-Tev expression plasmid (44). PCR amplification primers included an extra 15-nucleotide sequence at the 5′ end with homology to the cloning site of the pET plasmid (see Table S1 for the sequences of all primers). Primers Galp Mutase PIPE S and Galp Mutase PIPE AS were used to amplify the *bcugm* gene. For the vector, primers ZL169 and KB_T7t were used to amplify the pET28b-TEV which when expressed gives a fusion protein with N-terminal six histidines followed by a tobacco etch virus (TEV) recognition amino acid sequence.

10.1128/mBio.01559-17.8 TABLE S1  Primers used in this study. Download TABLE S1, DOCX file, 0.01 MB. Copyright © 2017 Ma et al. 2017 Ma et al. This content is distributed under the terms of the Creative Commons Attribution 4.0 International license.

*Escherichia coli* Rosetta2(DE3)pLysS strains containing pET28b-TEV_BcUGM or pET28b-TEV_BcDH (a control plasmid) were cultured overnight at 37°C with shaking at 250 rpm in 5 ml LB medium supplemented with 50 μg/ml kanamycin and 35 μg/ml chloramphenicol. Each culture was transferred to a flask containing 250 ml LB medium supplemented with the same antibiotics, and the flasks were incubated (37°C, 250 rpm) until cell density reached an optical density at 600 nm (OD_600_) of 0.6 to 0.8. Isopropyl-β-d-1-thiogalactopyranoside (IPTG) was added to a final concentration of 1 mM, and the cultures were grown for 20 h (18°C, 250 rpm). The cells were collected by centrifugation (6,000 × *g*, 10 min at 4°C) and washed with 20 ml cold double-distilled water (DDW), and each cell pellet was resuspended in 10-ml cold lysis buffer (50 mM Tris-HCl [pH 7.5], 50 mM NaCl, 10% glycerol, 1 mM EDTA, 0.5 mM phenylmethylsulfonyl fluoride [PMSF], and 1 mM dithiothreitol [DTT]). The cells were lysed by sonication in ice-cold water using a Misonix S-4000 sonicator (Misonix, Inc., Farmingdale, NY) equipped with 1/8-in. microtip probe (12 cycles with 1 cycle consisting of a 20-s pulse at 30% amplitude and 30 s off). After sonication, the samples were centrifuged (6,000 × *g* for 15 min at 4°C), and the supernatant was recovered and centrifuged again at 20,000 × *g* for 30 min at 4°C. The resultant supernatant was subjected to Ni column purification using a Fast-Flow Ni^2+^-Sepharose column (GE Healthcare) (2 ml of resin packed in a polypropylene column [1-cm inner diameter by 15 cm]). Recombinant proteins were eluted with elution buffer containing increased concentrations of imidazole (10 to 250 mM) and analyzed by measurement of their specific activity and by SDS-PAGE followed by Coomassie blue staining.

### Enzyme activities and inhibition assay.

Activity of recombinant BcUGM was tested in a 50-μl reaction solution containing 50 mM Tris-HCl (pH 7.5), 1 mM NADPH, 1 mM flavin adenine dinucleotide (FAD), and 1 mM UDP-galactopyranose. The initial reactions were performed at 30°C for 1 h and then inactivated by 98°C for 5 min. An equal volume of chloroform was added, the tubes were centrifuged for 5 min at 14,000 × *g*, and 30 μl of the upper aqueous phase was removed and mixed with 57 μl acetonitrile and 3 μl of 0.5 M ammonium acetate (pH 4.35). A portion (25 μl) of this mixture was used for hydrophilic interaction liquid chromatography (HILIC) by high-performance liquid chromatography with a UV detector (HPLC-UV) and another 25 μl was analyzed by liquid chromatography coupled to tandem mass spectrometry (LC-MS/MS) (10). The amount of product formed was determined by HPLC-UV.

Triplicate BcUGM assays were performed for kinetic analyses with increasing UDP-Gal*p* concentrations (0.1, 0.5, 1, 1.5, 2, and 3 mM). Reactions were carried out at 30°C for 30 min with 20 μl purified BcUGM with a fixed amount of FAD, NADPH, and Tris-HCl (pH 7.5). The amount of UDP-Gal*f* was analyzed by HPLC-UV and verified by liquid chromatography and mass spectrometry with electrospray ionization, ion trap, and time of flight technologies (LC-MS-ESI-IT-TOF). Kinetics were calculated by fitting enzyme kinetic curves using the GraphPad Prism 7 software.

For the inhibition assays, the reaction mixtures remained the same as for the kinetic analyses with the addition of various concentrations of UDP-KDG (1, 10, and 100 μM). To obtain UDP-KDG compound, a large-scale reaction was performed with *B. cinerea* UDP-glucose-4,6-dehydratase (BcDH) that converts UDP-glucose to UDP-KDG. This 1-ml reaction consisted of 50 mM Tris-HCl (pH 7.5), 1 mM UDP-glucose, 1 mM NAD^+^, and BcDH (14). The reaction was conducted for 2 h and heat inactivated, and the product was separated by HPLC-UV and verified by LC-MS-ESI-IT-TOF. Calculation of inhibition was performed using GraphPad Prism 7.

### Generation of *B. cinerea* mutant strains.

*B. cinerea ugm* homologue (*bcugm*) (GenBank accession number XM_001551972) was identified by a BLAST search with *Aspergillus fumigatus* UDP-galactopyranose mutase (GenBank accession number AJ871145). The NCBI *B. cinerea* B05.10 genome sequence (GenBank accession number XM_001551972) and flanking region was used to design primers (Table S1).

*B. cinerea bcugm* deletion strains (Δ*bcugm* strains) were generated by replacing the entire open reading frame (ORF) of the *bcugm* gene with a hygromycin resistance cassette consisting of an *Aspergillus nidulans oliC* promoter (*PoliC*), a hygromycin phosphotransferase gene (*hph*), and *bcacr* (*B. cinerea* enoyl-[acyl carrier protein] reductase) terminator (*Tacr*) (45). A deletion cassette was generated by adding 500 bp each of the 5′ and 3′ flanking regions of the *bcugm* gene on either side of the *hph* cassette by overlap PCR (Fig. S4). A double deletion strain of the *bcer* and *bcugm* genes (Δ*bcer* Δ*bcugm* strain) was generated by deleting the *bcugm* ORF with a nourseothricin resistance cassette (*nr*) in the background of a Δ*bcer* strain (short for Δ*bcer9a2* strain as mentioned in reference 10. The *bcugm-nr* deletion construct was assembled in the same way as described for the *hph* deletion cassette (Fig. S5).

10.1128/mBio.01559-17.4 FIG S4  Generation of the Δ*ugm* deletion strain in *B. cinerea*. The deletion cassette used to transform the wild-type strain contained the hygromycin resistance gene (*hph*) flanked by two regions of the *ugm* gene. The positions of the PCR primers used to verify the transformants are indicated by arrows. Download FIG S4, TIF file, 2.4 MB. Copyright © 2017 Ma et al. 2017 Ma et al. This content is distributed under the terms of the Creative Commons Attribution 4.0 International license.

10.1128/mBio.01559-17.5 FIG S5  Deletion of *ugm* in the background of the Δ*er* strain to generate theΔ*er* Δ*ugm* double deletion strain. The deletion cassette used to transform the Δ*er* strain contained the nourseothricin resistance gene (*nr*) flanked by two regions of the *ugm* gene. The positions of the PCR primers used to verify the transformants are indicated by arrows. Download FIG S5, TIF file, 2.0 MB.. Copyright © 2017 Ma et al. 2017 Ma et al. This content is distributed under the terms of the Creative Commons Attribution 4.0 International license.

A  *bcugm* overexpression construct was prepared by placing the *bcugm* ORF under the strong H2B promoter of *B. cinerea* (10) and flanked by an *nr* selection cassette. The *B. cinerea*-specific *bcniiA* sequence (46) was included as a homology region for integration of the construct (pNAH-ugm) to the *bcniiA* locus in the *B. cinerea* genome (Fig. S6). The plasmid was linearized with ApaI restriction enzyme and used to transform the *B. cinerea* Δ*er* strain to generate the Δ*bcer*::OE*bcugm* strain as described previously (10).

10.1128/mBio.01559-17.6 FIG S6  Map of *B. cinerea ugm* overexpression plasmid pNAH-ugm. The *ugm* ORF was placed under the *B. cinerea* H2B promoter by insertion into the AscI and NotI sites of pNAH. Download FIG S6, TIF file, 2.6 MB. Copyright © 2017 Ma et al. 2017 Ma et al. This content is distributed under the terms of the Creative Commons Attribution 4.0 International license.

A  *bcugm* complementation cassette was prepared by assembling three PCR fragments using a Gibson Assembly master mix kit (New England Biolabs). The three fragments include the *bcugm* gene with the upstream 500-bp region as the promoter and the downstream 340 bp as the terminator, the PoliC-nr cassette, and the Tcel5a (*B. cinerea* cel5a; GenBank accession number AY618929.1) termination signal (Fig. S7). This construct was transformed to a Δ*bcugm* strain to generate the *bcugm* complementation strain (Δ*bcugm*+*bcugm* strain).

10.1128/mBio.01559-17.7 FIG S7  The *ugm* complementation cassette. The *ugm* complementation cassette contains the intact *ugm* gene and the nourseothricin resistance gene (*nr*). Download FIG S7, TIF file, 1.6 MB. Copyright © 2017 Ma et al. 2017 Ma et al. This content is distributed under the terms of the Creative Commons Attribution 4.0 International license.

Genetic transformation of *B. cinerea* with the different DNA constructs was performed as previously described (10). Colonies that developed on the selection media were transferred to separate plates. At least 10 isolates were obtained for each type of strain, and DNA was extracted from each colony and analyzed by PCR to verify integration of the construct at the desired locus. Homokaryotic strains were obtained by single-spore isolation using a Sporeplay dissection microscope (Singer Instruments, UK). Derived colonies were analyzed by PCR to verify that the strain is homokaryotic at the desired locus, and additional rounds of single spore isolation were performed in cases of impurity. At least four separate single-spore isolates were obtained for each strain.

### Cell wall integrity assay.

Spores from 9-day-old PDA cultures were collected using sterile DDW and filtered through two layers of Miracloth (Millipore, USA). The spores were then diluted to 10^5^ spores/ml, and 5 μl of a spore suspension of each strain was applied to the center of a PDA plate without or with the specified concentration of cell wall-inhibiting compounds calcofluor white (CW) or Congo red (CR). The plates were incubated at 22°C, and the radius of each colony was measured 3 days postinoculation (dpi). Each experiment was repeated at least five times with three replications per treatment.

To measure the MIC of caspofungin, the filtered spores were mixed into 20 ml of 50°C GB5-Glc medium to a final concentration of 10^4^/ml and immediately poured in 9-cm-diameter petri plates. Next, the Etest strips (bioMérieux, France), containing a continuous caspofungin concentration gradient from 0.002 to 32 μg/ml, were placed on the surface of the inoculated agar media and incubated at 22°C. The Etest MIC was defined as the drug concentration at which the border of the elliptical zone of complete inhibition intersected the scale on the antifungal test strip. Assays were repeated three times.

### Pathogenicity assay.

Pathogenicity assays were performed on the leaves of 10-day-old bean plants (*Phaseolus vulgaris* cv. Dwarf sugar) as previously described (47). Four leaves from two plants (two leaves per plant) were used for each treatment. Leaves were inoculated with 7.5-μl droplets of spore suspension (10^5^ spores/ml in GB5-Glc), and the radius of lesions was recorded every 24 h. Experiments were repeated at least five times.

### Isolation of fungal proteins. (i) Cell wall proteins.

Spores were collected from 9-day-old PDA cultures, filtered through two layers of Miracloth, and then added to a 50-ml Erlenmeyer flask with 10 ml PDB to a final concentration of 10^6^ spores/ml. Samples were incubated on an orbital shaker with agitation at 180 rpm for 24 h. Then, samples were centrifuged at 3,000 × *g* for 10 min, and the supernatant was removed. Mycelial pellets were washed sequentially with 20 ml of DDW, 20 ml of washing solution A (1 M NaCl, 25 mM EDTA, 1 mM PMSF), and 20 ml of lysis buffer (10 mM Tris-HCl [pH 7.4], 10 mM EDTA, 1 mM PMSF) to remove proteins present in the medium and proteins that are loosely connected with the hyphae. This sequential washing cycle was performed three times for each washing step, then the washed mycelial pellets were dried by lyophilization overnight, and the dried pellets were homogenized by repeated homogenization using a TissueLyser (Qiagen) operating at 30 Hz for 3 min each time until complete cell breakage.

Cell wall proteins were isolated by a previously reported procedure (48) with modifications. The broken cells were suspended in 1 ml of ice-cold lysis buffer, vortexed thoroughly, and centrifuged at 1,200 × *g* at 4°C for 10 min, and then the supernatant was decanted carefully. The cell wall pellets were suspended and washed sequentially in 20 ml of wash solution B (5 mM EDTA, 1 mM PMSF), 20 ml of wash solution C (1 M NaCl, 5 mM EDTA, 1 mM PMSF), and 20 ml of wash solution D (50 mM Tris-HCl [pH 8.0], 10 mM EDTA, 1 mM PMSF), with centrifugation at 1,200 × *g* at 4°C for 10 min between each washing step. This sequential washing cycle was repeated three times for each washing step, the supernatant was decanted, and the wet wall pellets were weighed. Washed wall pellets were suspended in extraction buffer (50 mM Tris-HCl [pH 8.0], 50 mM EDTA, 2% [wt/vol] SDS supplemented with 30 mM 2-mercaptoethanol) at a ratio of 5 ml of buffer per 1 g of tissue, and the samples were vortexed for 10 s, boiled for 10 min, and then centrifuged at 12,000 × *g* for 10 min. The supernatant was retained and concentrated using Vivaspin 6 centrifugal concentrators (Sartorius, Germany) with 10-kDa molecular weight cutoff (MWCO) at 3,000 × *g* for 60 min. After every round of concentration, phosphate-buffered saline (PBS) buffer was added to the concentrator (Vivaspin 6) to rinse and remove excessive interference agents for protein quantification. The process was repeated six times, and protein concentration was determined using the bicinchoninic acid protein assay kit (Sigma).

### (ii) Total proteins.

Mycelia were collected, and the entire mycelial biomass was washed following the same steps as described above. The wet mycelial pellets were weighed and lyophilized, and then the dried frozen mycelia were homogenized and suspended in extraction buffer (5 ml per 1 g [wet weight] ofmycelia). Samples were boiled for 10 min and centrifuged at 12,000 × *g* for 10 min, and the supernatant containing total proteins was collected and subjected to Vivaspin concentration and bicinchoninic acid quantification as mentioned above.

### Western blot analysis.

Equal amounts of protein extracts were mixed with 4× SDS protein sample buffer (40% glycerol, 240 mM Tris-HCl [pH 6.8], 8% SDS, 0.04% bromophenol blue, 5% beta-mercaptoethanol), denatured by boiling for 5 min, and then separated on SDS-10% PAGE. Then, the gel was blotted onto a 0.45-μm nitrocellulose membrane (Bio-Rad Laboratories), using wet transfer buffer (25 mM Tris base, 190 mM glycine [pH 8.3]) at 100 V for 1 h. The membranes were stained briefly with 0.2% Ponceau S dye. The blots were incubated for 2 h in blocking solution containing both Tris-buffered saline with Tween 20 (TBST) (50 mM Tris-HCl [pH 7.5], 200 mM NaCl, 0.05% [vol/vol] Tween 20) and low-fat powdered milk reconstituted to 5% (wt/vol), and then incubated for 2 h at room temperature with culture supernatant of the hybridoma producing monoclonal antibodies L10-1 which is specific for galactofuranose epitopes (25) at a 1:5 dilution in blocking solution. Mouse antiactin monoclonal antibody (MAB1501; Chemicon) was used as a control and to determine protein amounts. The membranes were washed in TBST and then incubated for 1 h at room temperature with goat anti-mouse immunoglobulin G conjugated to horseradish peroxidase (Jackson ImmunoResearch, USA), diluted 1:2,000 in blocking solution. The membranes were washed four times for 5 min each time with TBST and then developed with 10 ml of enhanced chemiluminescence (ECL; Amersham) detection solutions for 1 min.

### RNA extraction and quantitative real-time PCR.

*B. cinerea* strains were cultured under the same growth condition used for the isolation of cell wall proteins, mycelia were harvested 24 h postinoculation (hpi) by filtration through Miracloth and immediately frozen in liquid nitrogen. For RNA extraction, frozen samples were crushed using a TissueLyser (Qiagen). Total RNA was then isolated using a NucleoSpin RNA plant kit (Macherey-Nagel), and residual DNA was digested with DNase I (Thermo Scientific). An aliquot (1 µg) of RNA was used as the template for cDNA synthesis with oligo(dT) primer using RevertAid first-strand cDNA synthesis kit (Thermo Scientific). Expression levels of *bcugm* were determined by real-time quantitative PCR (qPCR) with primers 24/25 (Table S1) using SYBR Premix *Ex Taq* II (TaKaRa) in an Mx3000P real-time PCR apparatus (Stratagene). The *bcgpdh* (BC1G_05277, primers 26/27) was used as a control gene to normalize data. The experiments were repeated three times.

### Spore germination assay.

Spores from 9-day-old PDA cultures were collected in PDB, filtered through two layers of Miracloth, and diluted to 5 × 10^4^/ml, and 30 μl of spore suspension was added to each well of a 24-well cell culture plate (SPL Life Sciences, South Korea). The plate was incubated at 22°C, and germination rates were determined at 2-h intervals using an inverted microscope (Olympus). Experiments included four replications with 50 ± 10 spores per sample. The experiments were repeated three times.

### Statistical analyses.

The statistical significance between means was tested by Student’s *t* test (an asterisk indicates *P* < 0.01 by two-tailed *t* test). In all graphs, values are the mean values for at least three independent experiments, each with at least three replications per treatment.
