# Supplementary figures and images for: UDP-4-Keto-6-Deoxyglucose, a Transient Antifungal Metabolite, Weakens the Fungal Cell Wall Partly by Inhibition of UDP-Galactopyranose Mutase
Source: mBio. 2017 Nov 21;8(6):e01559-17. doi: 10.1128/mBio.01559-17 (PMC5698552; doi:10.1128/mBio.01559-17)

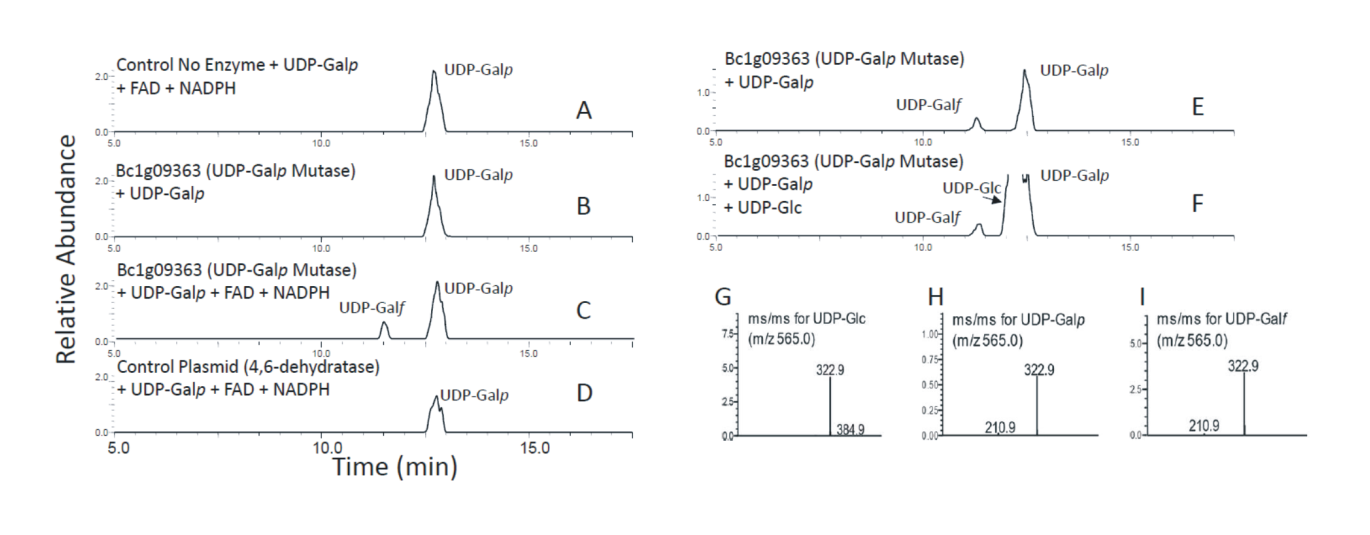

Supplement: FIG S1 [file mbo006173614sf1.tif]

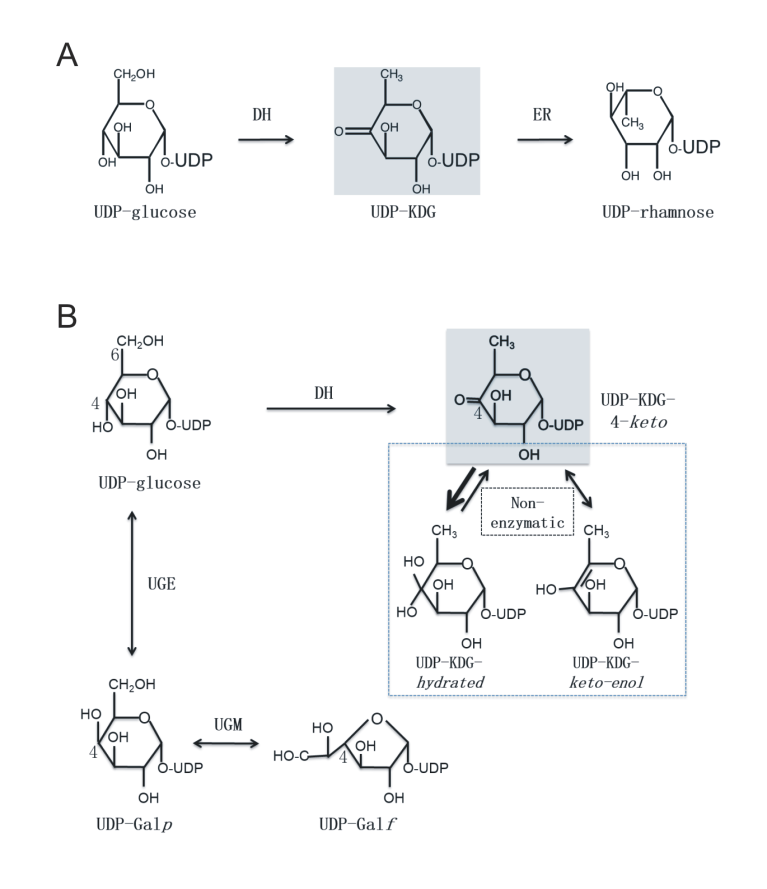

Supplement: FIG S2 [file mbo006173614sf2.tif]

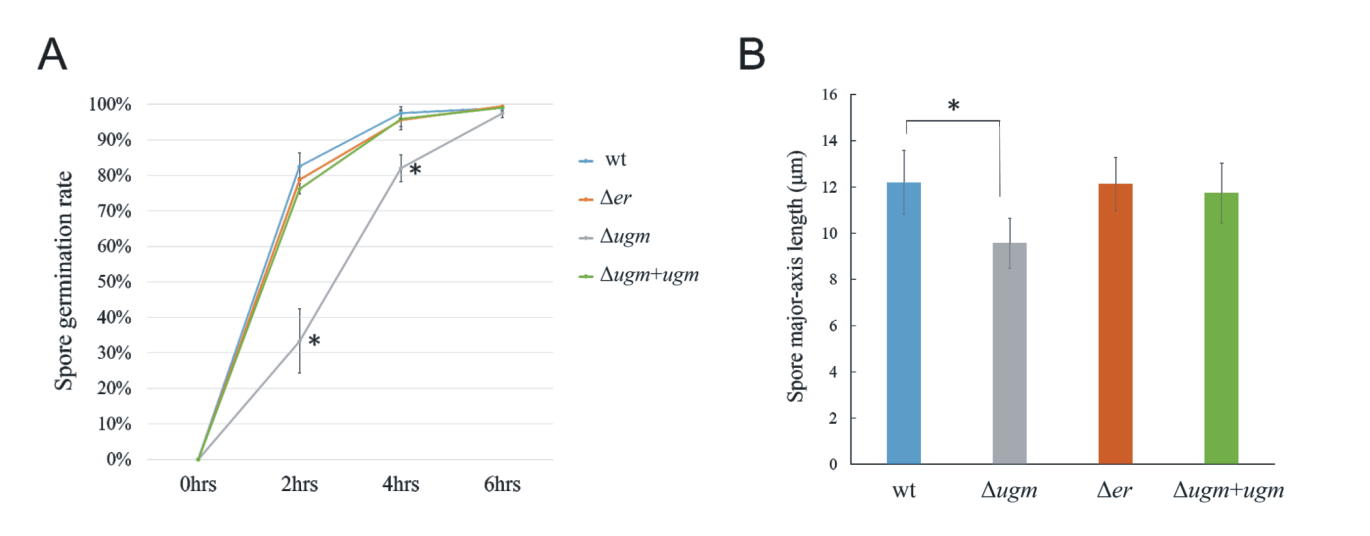

Supplement: FIG S3 [file mbo006173614sf3.tif]

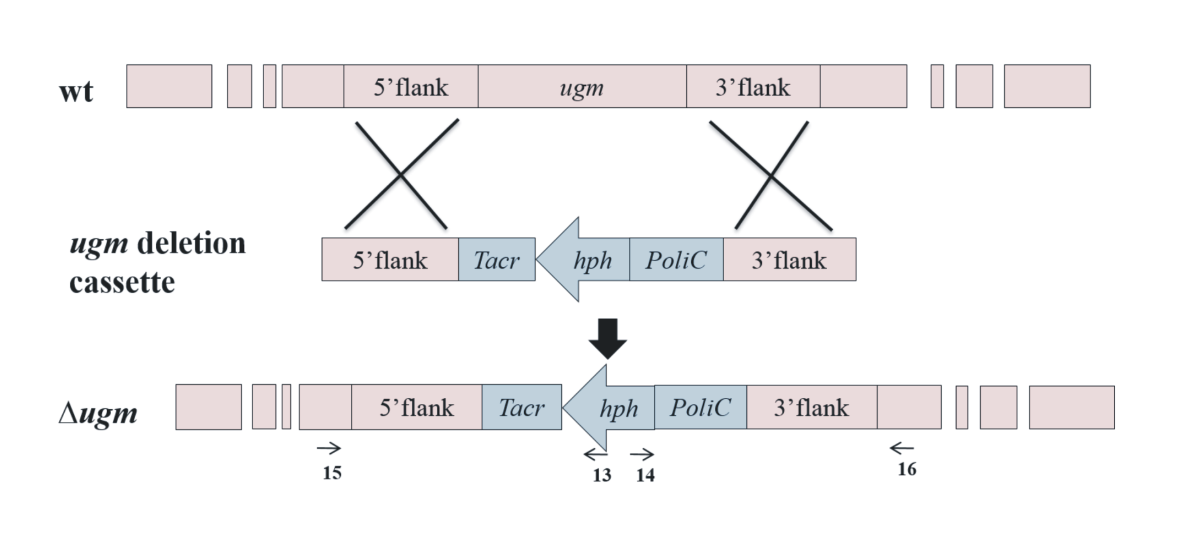

Supplement: FIG S4 [file mbo006173614sf4.tif]

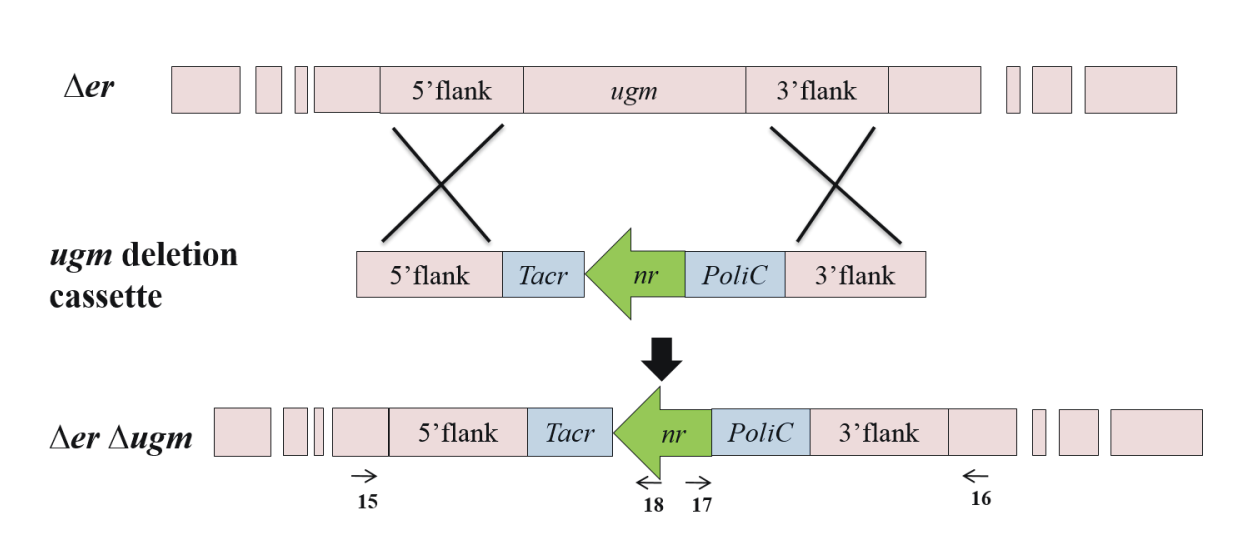

Supplement: FIG S5 [file mbo006173614sf5.tif]

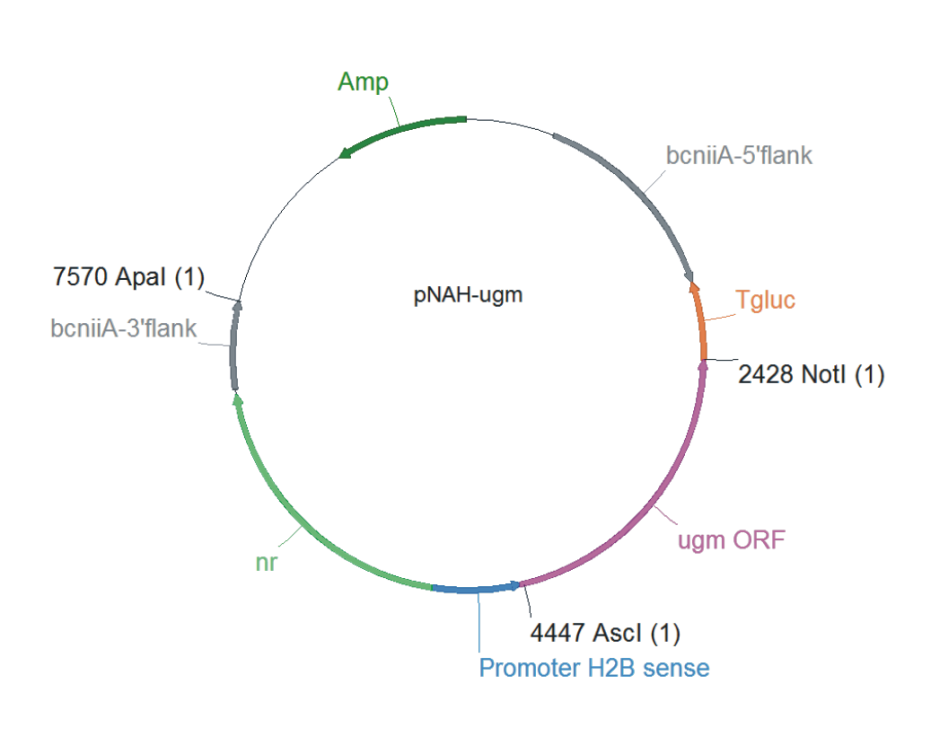

Supplement: FIG S6 [file mbo006173614sf6.tif]

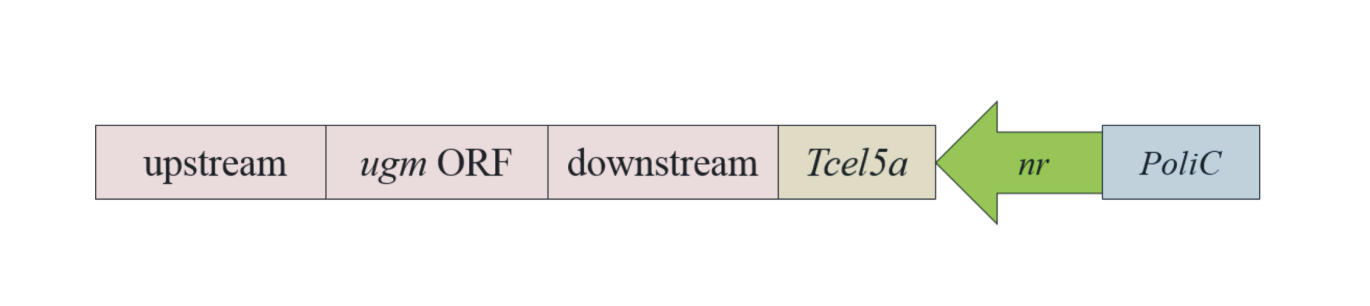

Supplement: FIG S7 [file mbo006173614sf7.tif]
