# Supplementary material for: UDP-4-Keto-6-Deoxyglucose, a Transient Antifungal Metabolite, Weakens the Fungal Cell Wall Partly by Inhibition of UDP-Galactopyranose Mutase
Source: mBio. 2017 Nov 21;8(6):e01559-17. doi: 10.1128/mBio.01559-17 (PMC5698552; doi:10.1128/mBio.01559-17)
Supplement: TABLE S1 [file mbo006173614st1.docx]

Table S1. Primers used in this study.

| Primer | Name | Purpose | Sequence (5’- 3’) |
| --- | --- | --- | --- |
| 1 | pTZ-5'BcGMfor | Amplify 5’flank of *bcugm* | GCCAGGGTTTTCCCAGTCACGACGCAAGGTTCGAGAATCGTCCT |
| 2 | hph-5'BcGMrev | Amplify 5’flank of *bcugm* | TTTTTCAGGAATTATTCTCACAGTCATACATGTCGGCCATTGTG |
| 3 | hph-3'BcGMfor | Amplify 3’flank of *bcugm* | GATGCATTCGCGAGGTACCGAGCTATGTGCCTTTAGGAGTTTGG |
| 4 | pTZ-3'BcGMrev | Amplify 3’flank of *bcugm* | TAACAATTTCACACAGGAAACAGCAGAGGCATGTGATCCTGACT |
| 5 | OliCpromHygpOnexfor | Amplify *hph* cassette | AGCTCGGTACCTCGCGAATGC |
| 6 | ToACRrev | Amplify *hph* cassette | ACTGTGAGAATAATTCCTG |
| 7 | nat-3'BcGMfor | Amplify nr cassette | AGCGAATTCGTAATCATGGTCATAATGTGCCTTTAGGAGTTTGG |
| 8 | Policfor | 5’ primer of *Polic* | TATGACCATGATTACGAATTC |
| 9 | NotI-BcGMrev | Amplify *bcugm* ORF to clone into pNAH | TTTGCGGCCGCCTAGTTGGCGAGACGTGCAG |
| 10 | AscI-BcGMfor | Amplify *bcugm* ORF to clone into pNAH | TTTGGCGCGCCGACATGTATGTATCCCCTTCTC |
| 11 | XhoI-BcGM | Check the remaining of *bcugm* | CCCTCGAGTACGATGTTGGCGGACACGT |
| 12 | Hind3-BcGM | Check the remaining of *bcugm* | CCCAAGCTTGGAACTGCCCAGACCTTGT |
| 13 | Hyg end for | Used to check homologous recombination of *hph*-containing cassette | ATTCCCAATACGAGGTCGCCAACATCTTCTTC |
| 14 | Hyg beg rev | Used to check homologous recombination of *hph*-containing cassette | GAGATGCAATAGGTCAGGCTCTCGCTGAATTC |
| 15 | BcGM-5'check for | Used to check *bcugm* homologous recombination | CGTGTGAGTAAAGGAGTGGC |
| 16 | BcGM-3'check rev | Used to check *bcugm* homologous recombination | ACTAGATCCAGGCATTACAC |
| 17 | nat end rev | Used to check homologous recombination of *nr*-containing cassette | TAAGCCGTGTCGTCAAGAG |
| 18 | NAT_ORF_end_for | Used to check homologous recombination of *nr*-containing cassette | TGAGCGGCCTGCAGGAATTCTAGAG |
| 19 | bcniiA-hi5F | Check 5’ homologous recombination of pNAH-ugm into *bcniiA* locus | GCGGGGTATGGCAGCATGAGTG |
| 20 | Tgluc-hiF | Check 5’ homologous recombination of pNAH-ugm into *bcniiA* locus | CATACGTACATCTGATTTGACAACC |
| 21 | bcniiA-hi3R | Check 3’ homologous recombination of pNAH-ugm into *bcniiA* locus | CTTATAGCAAGCGCGATGTGTATC |
| 22 | Tglu rev Hind3 | Amplify cel5a terminator for incorporating it to *nr* cassette | CCCAAGCTTTACCCCACGATCTTGTTGGG |
| 23 | Tglu-3'ugm | Amplify ugm to fuse it to *nr* cassette | AGATCGTGGGGTAAAGCTTGGGCTACCCTCAACAATCACCAG |
| 24 | RT-GMfor | For Real-time PCR amplification of *bcugm* | GGTGTTGGAATTAGAGGAGAGCGAC |
| 25 | RT-GMrev | For Real-time PCR amplification of *bcugm* | TCTCAGGTTGGTTGTATGGGGAGT |
| 26 | rt-gapdh for | For Real-time PCR amplification of *bcgpdh* | CGAAGAATAGCACAAACAGCTGGAC |
| 27 | rt-gapdh rev | For Real-time PCR amplification of *bcgpdh* | CGTCACCTTATGCTTCTTGCTCC |
| 28 | Galp Mutase PIPE S | Amplify *bcugm* to be cloned into pET | TTTCAGGGCGCCATGGCGGATATCAACGTGGATGTG |
| 29 | Galp Mutase PIPE AS | Amplify *bcugm* to be cloned into pET | ATTGCTCAGCGGCTAATTCGCCAGACGCGCC |
| 30 | ZL169 | Amplify pET28b-TEV_galK15.1 | CATGGCGCCCTGAAAATACAGGTTTTCGCC |
| 31 | KB_T7t | Amplify pET28b-TEV_galK15.1 | TAGCCGCTGAGCAATAACTAGCATAACCCCTTG |
